# Persistent left superior vena cava

**DOI:** 10.5830/CVJA-2016-084

**Published:** 2017

**Authors:** Kamil W Tyrak, Mateusz K Hołda, Mateusz Koziej, Katarzyna Piątek, Wiesława Klimek-Piotrowska

**Affiliations:** Department of Anatomy, Jagiellonian University Medical College, Cracow, Poland; Department of Anatomy, Jagiellonian University Medical College, Cracow, Poland; Department of Anatomy, Jagiellonian University Medical College, Cracow, Poland; Department of Anatomy, Jagiellonian University Medical College, Cracow, Poland; Department of Anatomy, Jagiellonian University Medical College, Cracow, Poland

**Keywords:** coronary sinus, persistent left superior vena cava, right atrium, left atrium

## Abstract

Persistent left superior vena cava (PLSVC) is the most common congenital malformation of thoracic venous return and is present in 0.3 to 0.5% of individuals in the general population. This heart specimen was dissected from a 35-yearold male cadaver whose cause of death was determined as non-cardiac. The heart was examined and we found a PLSVC draining into the coronary sinus. The right superior vena cava was present with a small-diameter ostium. An anomalous pulmonary vein pattern was observed; there was a common trunk to the left superior and left inferior pulmonary veins (diameter 17.8 mm) and an additional middle right pulmonary vein (diameter 2.7 mm) with two classic right pulmonary veins. The PLSVC draining into the coronary sinus had led to its enlargement, which could have altered the cardiac haemodynamics by significantly reducing the size of the left atrium and impeding its outflow via the mitral valve.

## Introduction

Persistent left superior vena cava (PLSVC) is the most common congenital malformation of the thoracic venous return and is present in 0.3 to 0.5% of individuals in the general population with a normal heart, and 4.5% in individuals with congenital heart diseases.^1^ A PLSVC co-occurs with the right superior vena cava in 80 to 90% of cases,^2^ and may also be accompanied by other heart abnormalities, such as anomalous connections of the pulmonary veins, aortic coarctation, tetralogy of Fallot, transposition of the great vessels as well as dextroversion.^1^,^3^,^4^ Moreover, cardiac rhythm disturbances concerning impulse formation and conduction have been observed.

The PLSVC usually drains into the right atrium (in 80–92%) through a dilated coronary sinus (CS),^5^,^6^ but in approximately 10 to 20% of cases, it is associated with left atrial (LA) drainage.^7^,^8^ The PLSVC may drain directly through the left atrium or via the unroofed CS, which is a cause of right-to-left cardiac shunt. The majority of patients with PLSVC are asymptomatic. In general, only patients with unusual drainage and right-to-left shunting are of clinical significance. Anomalous venous return via the PLSVC may be the cause of cardiac arrhythmias, decreased exercise tolerance, progressive fatigue, chest discomfort, palpitations, syncope or cyanosis.^6^

The implications of existing PLSVC could be important for clinicians who are involved in placement of central venous-access devices.^9^ Access to the right side of the heart or pulmonary vasculature through the left subclavian vein is much more difficult in patients with PLSVC. Placement of a central line or cardiac resynchronisation therapy leads and pacemaker implantation in undiagnosed cases with PLSVC can result in incorrect positioning.^10^ In those cases, access to the right heart and coronary sinus should be performed via the right subclavian vein, allowing for an easier route. Also the presence of PLSVC is a relative contraindication to the administration of retrograde cardioplegia during cardiac surgery.^6^

## Case report

This heart specimen was dissected from a 35-year-old male cadaver (BMI 29.9 kg/m^2^) whose cause of death was determined as non-cardiac during a routine forensic autopsy. The heart weight was 613 g.

**Fig. 1.**
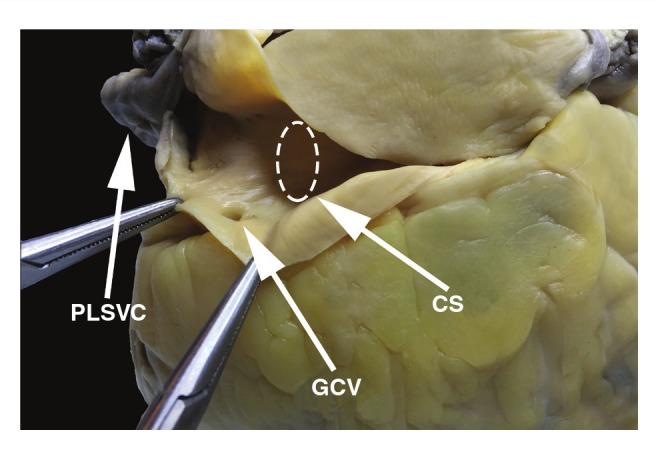
The persistent left superior vena cava drains into the coronary sinus in this heart specimen. CS, coronary sinus; GCV, great cardiac vein; PLSVC, persistent left superior vena cava.

After a month of fixation in 10% buffered formaldehyde, the heart was examined and it revealed PLSVC drains into the CS (Fig. 1). The mediolateral (ML) and anteroposterior (AP) diameters of the PLSVC, measured 1 cm above its connection with the CS, were 12.2 and 11.5 mm, respectively. The mean thickness of the LSVC was 0.6 mm. Further examination revealed an enormous coronary sinus with a funnel-shaped expansion at the PLSVC orifice. The CS diameter, measured in the middle of the structure, was greatly enlarged (15.85 mm). The CS ostium was also enlarged, measuring 17.2 mm in diameter.

**Fig. 2.**
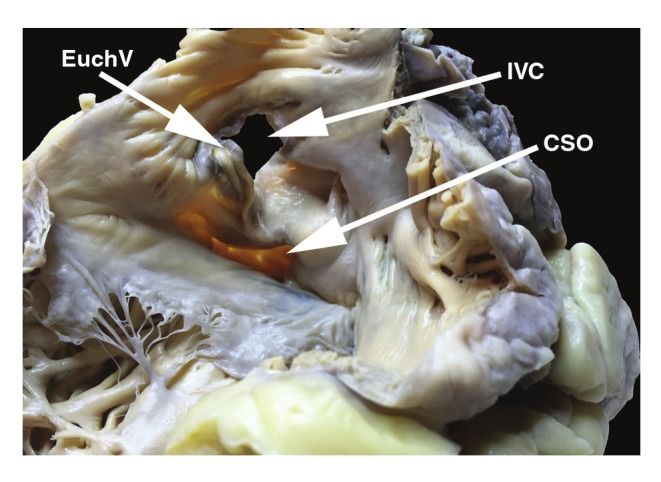
View of the internal surface of the right atrium. CSO, coronary sinus ostium; EuchV, Eustachian valve; IVC, inferior vena cava.

**Fig. 3.**
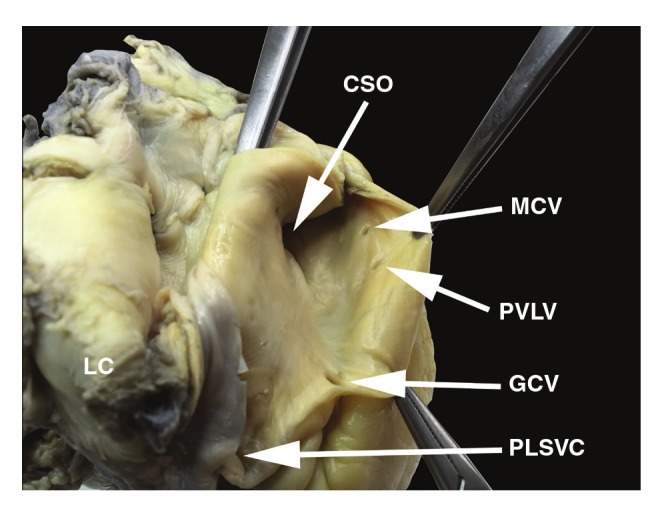
View of the internal surface of the ostia of the coronary sinus and its main tributaries. CSO, coronary sinus ostium; GCV, great cardiac vein; MCV, middle cardiac vein; PLSVC, persistent left superior vena cava; PVLV, posterior vein of the left ventricle.

The CS ostium valve (Thebesian valve) was absent (Fig. 2). The great cardiac vein had a relatively small ostium (diameter 2.3 mm) and lacked a Vieussens valve (Fig. 3). Other venous valves were also absent within the ostia of the middle cardiac vein and posterior vein of the left ventricle (diameter of veins < 1 mm). The small cardiac vein was absent. The length of the CS, as measured from the ostium of the great cardiac vein to the CS orifice, was 43.7 mm. The right superior vena cava was present with a small ostium diameter (ML = 14.3 mm; AP = 14.9 mm).

Distortions of the atrial dimensions were noted; reduction in the AP length of the left atrium and enlargement of the right atrium. The dimensions of the atrioventricular rings were also measured; mitral ring (AP = 26.5 mm; ML = 12.4 mm; area = 2.6 cm^2^) and tricuspid ring (AP = 31.4 mm; ML = 21.6 mm; area = 5.3 cm^2^). The inferior vena cava ostium diameters were AP = 28.6 mm and ML = 33.8 mm. The Eustachian valve was present Fig. 2)).

**Fig. 4.**
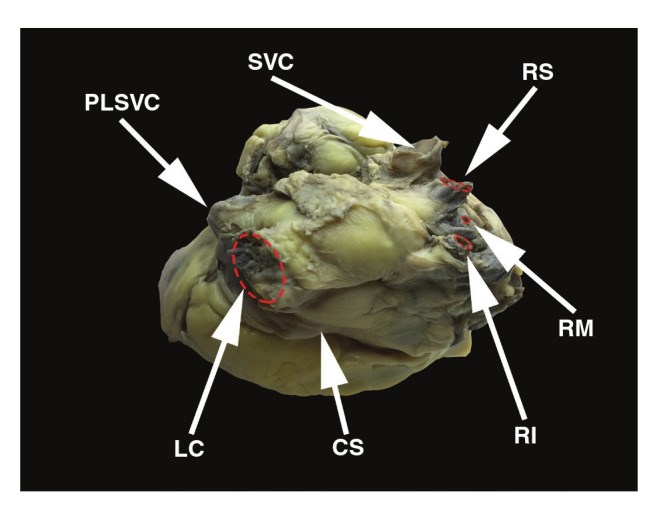
View of the posterior and superior wall of the left atrium. CS, coronary sinus; LC, common trunk of the left superior and left inferior pulmonary veins; PLSVC, persistent left superior vena cava; RI, right inferior pulmonary vein; RM, right middle pulmonary vein; RS, right superior pulmonary vein; SVC, superior vena cava.

**Fig. 5.**
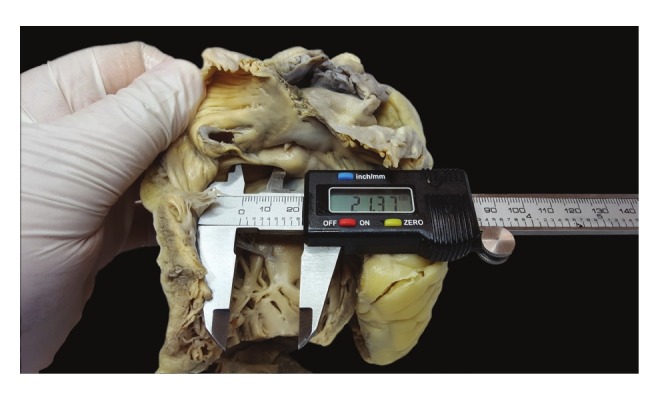
Measurements were performed with electronic calipers with 0.01-mm precision.

An anomaly of the pulmonary vein pattern was observed; there was a common trunk of the left superior and left inferior pulmonary veins (diameter 17.8 mm) and an additional middle right pulmonary vein (diameter 2.7 mm) with two classic right pulmonary veins (Fig. 4). The patent foramen ovale was absent and a left-sided septal pouch was observed.11 Fig. 5 shows how measurements were performed.

## Discussion

During embryogenesis, the sinus venosus consists of the right and left horns. Each receives blood from the common cardinal, vitelline and umbilical veins. During gestation, the left horn, after obliteration of the above veins, evolves into the coronary sinus and oblique vein of the left atrium, while the right becomes incorporated into the right atrium. The right common cardinal vein and the proximal part of the right anterior cardinal vein build the right superior vena cava. The left anterior cardinal vein changes into the internal jugular vein. The presence of the left anterior cardinal vein and obliteration of the left common cardinal vein leads to the formation of the left superior vena cava, which drains into the right atrium through the coronary sinus.^12^,^13^

The presence of a PLSVC has a significant influence on the anatomy of the heart and venous system. Although our autopsy study revealed enlargement of the heart chambers, the largest change concerned the CS. We compared the dimensions of the CS and selected heart structures from this case with mean dimensions obtained by measuring nearly 200 structurally normal hearts (23% female; mean age 46.7 ± 19.1 years) without PLSVC in our previous studies.^14^,^15^ The diameter of the CS described in this case report (17.17 mm) was the largest of all observed autopsy specimens, almost twice the average of all previous measurements (mean 9.2 ± 2.7 mm). The existence of a common left pulmonary vein trunk may also have been the result of the PLSVC, which limited the free space in the area where the left inferior pulmonary vein should be.^16^

The PLSVC drains about 20% of the whole venous return,^17^ and therefore significantly enhanced venous return via the CS forces an increase in its dimensions. Moreover, an increased blood volume flowing into the CS leads to the atrophy of the Vieussens, Thebesian and other heart vein valves. The enlargement of the CS is also often mentioned by other authors as the most characteristic change in the anatomy of the heart.

Furthermore, our observed changes were related to the size of the valves; the mitral valve area was substantially reduced (2.6 cm^2^; mean value 4.2 ± 1.8 cm2), which may have been an outcome of the pressure exerted by an enlarged CS on the left atrium and mitral ring. The tricuspid valve area (5.3 cm^2^) did not differ significantly compared to the average value of 4.8 ± 1.6 cm^2^. Venous return via the right superior vena cava was reduced, due to blood draining from the left arm, neck and head via the PLSVC. These haemodynamic effects explain the reduced dimensions of the right superior vena cava; AP = 17.3 mm and ML = 16.5 mm (mean 20.1 ± 3.6 mm and 18.3 ± 3.4 mm, respectively). Also the weight of the heart (613 g) showed an increase in comparison with the average value of 432.7 ± 112.8 g, with no cause of heart enlargement other than the PLSVC.

General and specific haemodynamic effects from the presence of the PLSVC vary between cases and depend largely on the coexistence of other heart abnormalities. The presence of a PLSVC influences mainly the blood flow in the atria and cardiac venous system. Patients with PLSVC are mainly asymptomatic or minimally symptomatic.

The most common variant of this anomaly is as follows; the PLSVC drains into the right atrium with a right superior vena cava present, which usually does not cause significant haemodynamic changes and clinical consequences. However, if the PLSVC drains into the left atrium, right-to-left cardiac shunt with desaturation and cyanosis as a consequence, is observed. The latter case often needs surgical intervention.^1^,^18^

Other complications resulting from the existence of PLSVC include difficulty in pulmonary artery catheterisation,^10^ cerebral abscess,^19^ arrhythmia and thromboembolic events^20^ or difficulties in left-sided right heart and cardiac venous system catheterisation.^21^ Enlarged CS (exerting pressure on the atrioventricular node) or absence of the right superior vena cava are common causes of cardiac rhythm disorders. The presence of a huge CS due to a persistent PLSVC can alter cardiac haemodynamics by significant reduction of the left atrium size and impediment of its outflow via the mitral valve, as presented in this case.

A PLSVC often coexists with other congenital heart defects, therefore early detection of this anomaly may be very important for the future outcome of the patient.^17^ Isolated PLSVC without a superior vena cava is very rare (0.1% of the population).^22^,^23^ In the majority of known cases, the CS is unroofed, which causes a right-to-left shunt of blood.^17^ In 10 to 20% of patients, the PLSVC drains into the left atrium, which may be associated with more dangerous haemodynamic complications. It is also worth mentioning that the incidence of defects in foetuses is higher than in the general population. This is due to the double mechanism: anatomical anomalies may cause spontaneous miscarriage, as well as the existence of PLSVC along with other heart defects may lead to premature death.^12^

Although diagnosis is not very complicated, the anomaly often remains unnoticed, especially when it is clinically inaudible. PLSVC is very often discovered accidentally during invasive cardiac procedures, mostly during routine left-sided right-heart catheterisation, surgical procedures or insertion of a venous central line.^21^,^24^ The presence of PLSVC can result in leftsided heart obstruction, which can cause a decrease in heart compliance and as a result, lower stroke volume.^25^

On chest X-ray, PLSVC can be seen as a widened shadow of the aorta with a visible venous half-moon shadow from the left side of the aortic arch to the middle of the left clavicle. Basic diagnostic methods include transoesophageal and transthoracic echocardiography. Other commonly used methods comprise conventional contrast venography, computed tomography and magnetic resonance venography.^17^ Prenatal diagnosis is based on echocardiography and mostly reveals an enlargement of the CS.^12^

## Conclusions

We present a case in which the PLSVC significantly affected anatomical relationships and dimensions of the heart. The PLSVC draining into the CS led to its enlargement and to atrophy of the Vieussens and Thebesian valves. The huge CS could have altered cardiac haemodynamics with a significant reduction in the size of the left atrium and impediment of its outflow via the mitral valve. Also the drainage of the pulmonary vein into the left atrium may have been affected due to the presence of the PLSVC.

## References

[1] Zhong YL, Long X-M, Jiang L-Y, He B-F, Lin H, Luo P (2015). Surgical treatment of dextroversion, isolated persistent left superior vena cava draining into the left atrium.. J Card Surg.

[2] Ruano, CA, Marinho-da-Silva A, Donato P (2015). Congenital thoracic venous anomalies in adults: morphologic MR imaging.. Curr Probl Diagn Radiol.

[3] Kula S, Cevik A, Sanli C, Pektas A, Tunaoglu FS, Oguz AD (2011). Persistent left superior vena cava: experience of a tertiary health-care center.. Pediatr Int.

[4] Buirski G, Jordan SC, Joffe HS, Wilde P (1986). Superior vena caval abnormalities: their occurrence rate, associated cardiac abnormalities and angiographic classification in a paediatric population with congenital heart disease.. Clin Radiol.

[5] Granata A, Andrulli S, Fiorini F, Logias F, Figuera M, Mignani R (2009). Persistent left superior vena cava: what the interventional nephrologist needs to know.. J Vasc Access.

[6] Goyal SK, Punnam SR, Verma G, Ruberg FL (2008). Persistent left superior vena cava: a case report and review of literature.. Cardiovasc Ultrasound.

[7] Dinasarapu CR, Adiga GU, Malik S (2010). Recurrent cerebral embolism associated with indwelling catheter in the presence of anomalous neck venous structures.. Am J Med Sci.

[8] Uçar O, Paşaoğlu L, Ciçekçioğlu H, Vural M, Kocaoğlu I, Aydoğdu S (2010). Persistent left superior vena cava with absent right superior vena cava: a case report and review of the literature.. Cardiovasc J Afr.

[9] Povoski SP (2000). A prospective analysis of the cephalic vein cutdown approach for chronic indwelling central venous access in 100 consecutive cancer patients.. Ann Surg Oncol.

[10] Lai YC, Goh JC, Lim SH, Seah TG (1998). Difficult pulmonary artery catheterization in a patient with persistent left superior vena cava.. Anaesth Intensive Care.

[11] Hołda M, Koziej M, Hołda J, Piątek K, Tyrak K, Chołopiak W (2016). Atrial septal pouch – morphological features and clinical considerations.. int J Cardiol.

[12] Pasquini L, Belmar C, Seale A, Gardiner HM (2006). Prenatal diagnosis of absent right and persistent left superior vena cava.. Prenat Diagn.

[13] Miraldi F, di Gioiga CR, Proietti P, De Santis M, d'Amati G, Gallo P (2002). Cardinal vein isomerism: an embryological hypothesis to explain a persistent left superior vena cava draining into the roof of the left atrium in the absence of coronary sinus and atrial septal defect.. Cardiovasc Pathol.

[14] Hołda MK, Klimek-Piotrowska W, Koziej M, Mazur M (2015). Anatomical variations of the coronary sinus valve (Thebesian valve): implications for electrocardiological procedures.. Europace.

[15] Klimek-Piotrowska W, Hołda MK, Koziej M, Piątek K, Hołda J (2016). Anatomy of the true interatrial septum for transseptal access to the left atrium.. Ann Anat, Anatomischer Anzeiger.

[16] Klimek-Piiotrowska W, Hołda MK, Piątek K, Koziej M, Hołda J (2016). Normal distal pulmonary vein anatomy.. Peer J.

[17] Povoski SP (2011). Persistent left superior vena cava: review of the literature, clinical implications, and relevance of alterations in thoracic central venous anatomy as pertaining to the general principles of central venous access device placement and venography in cancer patients.. World J Surg Oncol.

[18] Lenox CC, Zuberbuhler JR, Park SC, Neches WH, Mathews RA, Fricker FJ (1980). Absent right superior vena cava with persistent left superior vena cava: implications and management.. Am J Cardiol.

[19] Lee MS, Pande RL, Rao B, Landzberg MJ, Kwong RY (2011). Cerebral abscess due to persistent left superior vena cava draining into the left atrium.. Circulation.

[20] Sarodia BD, Stoller JK (2000). Persistent left superior vena cava: case report and literature review.. Respir Care.

[21] Elison B, Evans D, Zanders T, Jeanmonod R (2014). Persistent left superior vena cava draining into the pulmonary venous system discovered after central venous catheter placement.. Am J Emerg Med.

[22] Erdoğan M, Karakaş P, Uygur F, Meşe B, Yamak B, Bozkir MG (2007). Persistent left superior vena cava: the anatomical and surgical importance.. West Indian Med J.

[23] Heye T, Wengenroth M, Schipp A, Dengler JT, Grenacher L, Kauffmann WG (2007). Persistent left superior vena cava with absent right superior vena cava: morphological CT features and clinical implications.. Int J Cardiol.

[24] luckianow G, Cole D, Kaplan L (2009). Anatomical variant found during catheter insertion.. J Am Acad Phys Assist.

[25] Liu X, He Y, Tian Z, Rychik J (2016). Persistent left superior vena cava connected to the coronary sinus in the fetus: effects on cardiac structure and flow dynamics.. Pediatr Cardiol.

